# The effect of proteolytic enzymes and pH on GII.4 norovirus, during both interactions and non-interaction with Histo-Blood Group Antigens

**DOI:** 10.1038/s41598-020-74728-z

**Published:** 2020-10-21

**Authors:** Manon Chassaing, Maëlle Robin, Julie Loutreul, Didier Majou, Gaël Belliot, Alexis de Rougemont, Nicolas Boudaud, Christophe Gantzer

**Affiliations:** 1 Food Safety Department, Actalia, 50000 Saint-Lô, France; 2 University of Lorraine, CNRS, LCPME, 54000 Nancy, France; 3 ACTIA, 75231 Paris Cedex 05, France; 4 Laboratory of Virology, National Reference Centre for Gastroenteritis Viruses, University Hospital of Dijon, 21000 Dijon, France; 5 UMR PAM A 02.102 Procédés Alimentaires et Microbiologiques, Université de Bourgogne Franche-Comté/AgroSup Dijon, 21000 Dijon, France

**Keywords:** Virus-host interactions, Gastroenteritis

## Abstract

Human noroviruses (HuNoVs) are the leading cause of acute gastroenteritis worldwide. Histo-Blood Groups Antigens (HBGAs) have been described as attachment factors, promoting HuNoV infection. However, their role has not yet been elucidated. This study aims to evaluate the ability of HBGAs to protect HuNoVs against various factors naturally found in the human digestive system. The effects of acid pH and proteolytic enzymes (pepsin, trypsin, and chymotrypsin) on GII.4 virus-like particles (VLPs) and GII.4 HuNoVs were studied, both during interactions and non-interaction with HBGAs. The results showed that GII.4 VLPs and GII.4 HuNoVs behaved differently following the treatments. GII.4 VLPs were disrupted at a pH of less than 2.0 and in the presence of proteolytic enzymes (1,500 units/mL pepsin, 100 mg/mL trypsin, and 100 mg/mL chymotrypsin). VLPs were also partially damaged by lower concentrations of trypsin and chymotrypsin (0.1 mg/mL). Conversely, the capsids of GII.4 HuNoVs were not compromised by such treatments, since their genomes were not accessible to RNase. HBGAs were found to offer GII.4 VLPs no protection against an acid pH or proteolytic enzymes.

## Introduction

Human noroviruses (HuNoVs) are a leading cause of acute gastroenteritis worldwide, with more than 700 million cases reported annually^1^. HuNoVs are transmitted through the faecal-oral route, and are among the main causes of viral foodborne outbreaks in both Europe and the United States of America (USA)^2,3^.

Noroviruses belong to the *Caliciviridae* family and can be divided into 10 genogroups (G): GI, GII, and GIV are responsible for most diseases in humans. In the GII genogroup, genotype 4 (GII.4) HuNoVs are the most prevalent type^4–7^. The capsid of about 30 nm have a major protein (VP1) and a minor protein (VP2). The external P2 subdomain of VP1 expresses the hypervariable region of the capsid. The capsid includes a single stranded RNA genome (~ 7.5 kilobases). VP1 can spontaneously assemble into virus-like particles (VLPs).

There is currently no available routine method for the detection of infectious HuNoVs in foodstuffs and the environment, although some innovative approaches to the in vitro replication of a few HuNoV genotypes have been described^8–10^. Reverse transcription–polymerase chain reaction (RT-qPCR) techniques are therefore currently used to detect and quantify HuNoVs in humans, foodstuffs, and the environment, in accordance with the ISO 15216 standard^11,12^.

It has been clearly demonstrated that HuNoVs interact with Histo-Blood Group Antigens (HBGAs) in humans^13–15^, and also with HBGA-like carbohydrates present in food, bacteria, and the environment^16^. HBGAs are complex carbohydrates resulting from the successive addition of monosaccharides to a precursor. The biosynthesis of HBGAs is governed by the ABO and *FUT2* and *FUT3* fucosyl-transferase gene families^17,18^. They can be found on red blood cells and mucosal epithelial cells. They can also be present as free antigens in biological fluids, such as saliva or milk^17,19^. The expression of active *FUT2* defines secretor status, while non-active *FUT2* defines non-secretor status^15,17^. Interactions between HuNoVs and HBGAs are highly specific, with multiple binding patterns and various affinities^18,20^. The binding of HuNoVs to HBGAs takes place in the P2 subdomain, which is composed of four or five highly conserved amino acids and a number of more variable amino acids^21,22^. Several studies have described HBGAs as attachment factors for several HuNoV strains that promote infection in humans, but it is still unclear whether they act as (co)-receptors or facilitate access to the infection site^8,15^.

Upon entry into the human body via oral ingestion, HuNoVs may (or may not) interact with salivary HBGAs, before passing through the gastrointestinal tract via the stomach. Thus, various factors may alter the structure of virions (i.e. infectious particles), especially the acid pH of the stomach and proteolytic enzymes in the gastrointestinal tract.

There is little data available on the resistance of HuNoVs as a function of pH. It has been observed that HuNoVs treated at pH 2.7 for 3 h were able to cause infection in human volunteers^23^. No reduction in HuNoV genomes was observed in Greenshell mussels at pH 3.8 following a 4-week incubation period^24^. To gain a better understanding of HuNoV survival, many studies have used cultivable infectious HuNoV surrogates, such as feline calicivirus (FCV), murine norovirus (MNV), tulane virus (TV), and VLPs. The combined results showed that a 30 min exposure to an acid pH ranging from 2.0 to 4.0 led to a 0–2 log_10_ reduction in these surrogates^25,26^, with the exception of FCV, which appeared to be more sensitive than the others^24,26,27^. The effect of pH on VLPs has been thoroughly reviewed and differences at the genogroup level have been highlighted, given that GII seemed more resistant to basic pH in the 8–10 range than GI^28,29^.

Other factors that can affect the structure of virions include proteolytic enzymes, whose effect is dependent on the nature of each enzyme. Pepsin enzymes, which are active at pH 1.0–3.0, may be present in the stomach and can hydrolyse the C-terminal of specific peptide bonds, such as phenylalanine, leucine, tyrosine, and tryptophan. Trypsin and chymotrypsin enzymes, which are active at pH 7.0–9.0, can be found in the duodenum and jejunum. Trypsin can hydrolyse peptide bonds located after an arginine or lysine residue, while chymotrypsin hydrolyses the C-terminal of peptide bonds, such as tyrosine, tryptophan, leucine, methionine, and phenylalanine. The different positive and negative effects of these enzymes on viruses have been described. Trypsin seems to promote HBGA-binding to the viral capsid of GII.3 and GII.6 VLPs^30,31^, while VP1 cleavage could be observed in the same genotypes^30–32^. Chymotrypsin and pepsin seemed to promote the infectivity of reovirus, while a reduction below 1 log_10_ in GI.1 and GII.4 HuNoV genomes could be observed after 42 days in a buffer mimicking the conditions of gastric fluid^33,34^. To our knowledge, no data are available regarding the influence of HBGA-binding on the potential protection or degradation of the viral capsid towards these inactivating factors found in the human digestive system.

The aim of this study was (1) to evaluate the effects of acid pH, pepsin, chymotrypsin, and trypsin on GII.4 VLPs and HuNoVs and, (2) investigate the potential protective effect of HBGA-binding on these viral particles, using the same parameters. The resulting effects on VLPs were analysed by examining capsid integrity as reflected in the overall structure of the particles (using transmission electron microscopy: TEM); measurements of particle size (using dynamic light scattering: DLS); protein mass modifications (using sodium dodecyl sulfate–polyacrylamide denaturing gel electrophoresis: SDS-PAGE); and conformational changes to the capsid (using a fluorescent hydrophobic probe: SYPRO Orange). The resulting effects on HuNoVs were evaluated using RNase treatment followed by RT-qPCR to estimate the number of genome copies (gc) from fully intact capsids^35^.

## Results

### Effects of acid pH and proteolytic enzymes on GII.4 VLPs

The effects of acid pH and proteolytic enzyme (pepsin, trypsin, and chymotrypsin) treatments at 37 °C for 4 h on the overall structure of GII.4 VLPs and the VP1 protein were evaluated.

**Figure 1:**
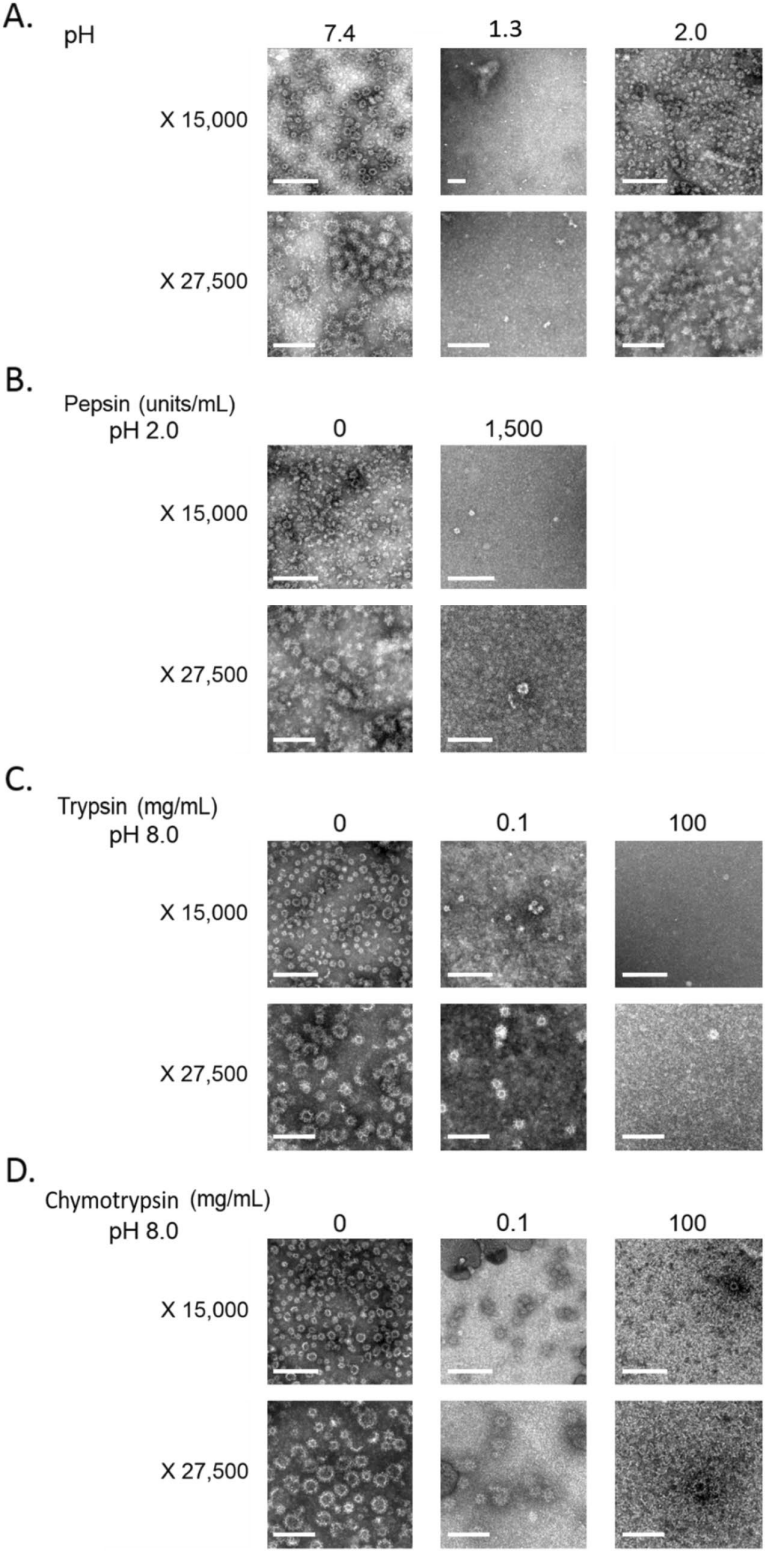
TEM observations of native and acid pH- or proteolytic enzyme-treated GII.4 VLP capsids after negative staining. **A.** GII.4 VLPs after exposure to pH 7.4, 1.3, and 2.0. **B.** GII.4 VLPs after exposure to 0 and 1,500 units/mL pepsin at pH 2.0. **C.** GII.4 VLPs after exposure to trypsin (0, 0.1, and 100 mg/mL) at pH 8.0. **D.** GII.4 VLPs after exposure to chymotrypsin (0, 0.1, and 100 mg/mL) at pH 8.0. The scale bar (solid line, bottom left corner) is 0.2 µm and 100 nm for X 15,000 and X 27,500 magnifications, respectively.

The overall structure of GII.4 VLPs was analysed using TEM, DLS, and SYPRO Orange probe. The study of conformational changes to the capsid using SYPRO Orange probe provided information about the melting temperatures (Tm values) of the particles. The results obtained by TEM are shown in Fig. 1. A magnification of ×15,000 was used to estimate the number of particles, while a magnification of ×27,500 was used to obtain a more precise estimation of capsid structure. Observations of the native GII.4 VLPs at pH 8.0 (i.e. control) showed a mean of around 70 particles per field. Two different sizes (20 and 35 nm) could be distinguished, expressing heterogeneous morphology. Particles of 20 and 35 nm are composed of 60 and 180 copies of VP1 respectively, and can be described as morphologically and antigenically similar^36^. Applying a pH of 3.0 or 2.0 did not change the mean number of particles per field, particle size, or morphology. At pH 1.3, the number of GII.4 VLPs decreased significantly, with no particles observed per field (unpaired sample *t*-test, *p* < 0.005) (Fig. 1A). Treatment with 1,500 units/mL pepsin at pH 2.0 also significantly reduced the number of GII.4 VLPs to a mean of two particles per field (unpaired sample *t*-test, *p* < 0.005) (Fig. 1B). After treatments with trypsin and chymotrypsin, the number of VLPs estimated by TEM decreased in a dose-dependent manner. With trypsin at pH 8.0, the mean numbers of particles per field were 70, 10, and 2 at 0, 0.1, and 100 mg/mL, respectively (unpaired sample *t*-test, *p* < 0.005). With chymotrypsin at pH 8.0, the mean numbers of particles per field were 70, 14, and 1 at 0, 0.1, and 100 mg/mL, respectively (unpaired sample *t*-test, *p* < 0.005). The mean size of particles was not modified after proteolytic enzyme treatments. All these results are summarized in Table 1, including results relating to the mean size of VLPs, measured by DLS, and the Tm values, determined using SYPRO Orange probe. For the pepsin treatment, only TEM was applied to define modifications on VLPs. The control shown in Table1 was valid for all conditions because there were no significant differences between them (unpaired sample *t*-test, *p* > 0.3). Interestingly, the mean size of particles either increased or decreased using DLS, depending on test conditions. The mean particle size was less than 6.4 nm after treatments with 100 mg/mL trypsin and chymotrypsin, and greater than 215.4 nm at pH 1.3. It ranged from 41.2 to 59.9 nm under the other conditions tested. This suggests that either GII.4 VLPs or their capsid proteins were unstructured or aggregated following proteolytic digestion (100 mg/mL) and at pH 1.3, respectively. The different mean sizes of isolated VLPs given by TEM and DLS are due to the fact that DLS measures the hydrodynamic diameter of the viral particles in aqueous media while TEM measures the physical diameter of the capsid after drying. On the other hand, when aggregation take place, DLS cannot detect particles over 6 µm and may be more sensitive to detect low numbers of particles between 200 nm and 6 µm compared to TEM. Tm value of the native VLPs at pH 8.0 was measured at 72.1 °C. No difference in the Tm values of the VLPs was observed at pH 2.0 or 3.0. The Tm value strongly decreased after the 0.1 mg/mL enzymatic treatments (unpaired sample *t*-test, *p* < 0.005), which means that proteolytic enzymes impacted the capsid structure, making it more vulnerable to heat. Tm values could not be estimated following the 100 mg/mL enzymatic treatments because of the high protein content caused by the addition of the enzymes.

**Table 1:** Number, size, and Tm of GII.4 VLPs after proteolytic enzyme treatments. The number, size, and Tm of GII.4 VLPs were determined using TEM, DLS, and SYPRO Orange probe, respectively.

Acid pH and proteolytic enzyme treatments		GII.4 VLPs			
		Number of particles (mean/field)	Mean size (nm)		Tm (°C)
		TEM	TEM	DLS	SYPRO
Control* 37 °C pH 8.0		70 ± 20	22.6 ± 7.7	50.3 ± 4.5	72.1 + / 0.3
Trypsin, pH 8.0 (mg/mL)	0.1	10^$^ ± 6	20.0 ± 3.2	51.1 ± 9.7	57.4^$^ ± 1.3
	100	2^$^ ± 1	21.0 ± 1.6	6.4^$^ ± 6.6	NA*
Chymotrypsin, pH 8.0 (mg/mL)	0.1	14^$^ ± 3	19.7 ± 2.3	41.2 ± 7.5	60.8^$^ ± 1.0
	100	1^$^ ± 1	21.0 ± 3.9	3.9^$^ ± 0.3	NA*
pH	1.3	0^$a^	NA***	215.4^$^ ± 33.5	NA**
	2.0	58 ± 6	20.7 ± 4.7	42.1 ± 4.0	72.1 ± 0.2
	3.0	72 ± 10	23.4 ± 6.4	59.9 ± 5.0	72.0 ± 0.2

*The control was valid for each condition tested. NA*: not measured because of the high protein content. NA**: not measured because of the absence of the fluorescence signal. NA***: not measured because of the absence of particles. ^a^0 means that no particle between 15 and 25 nm were observed. ^$^Unpaired Student’s *t*-test, *p* < 0.005.

**Figure 2:**
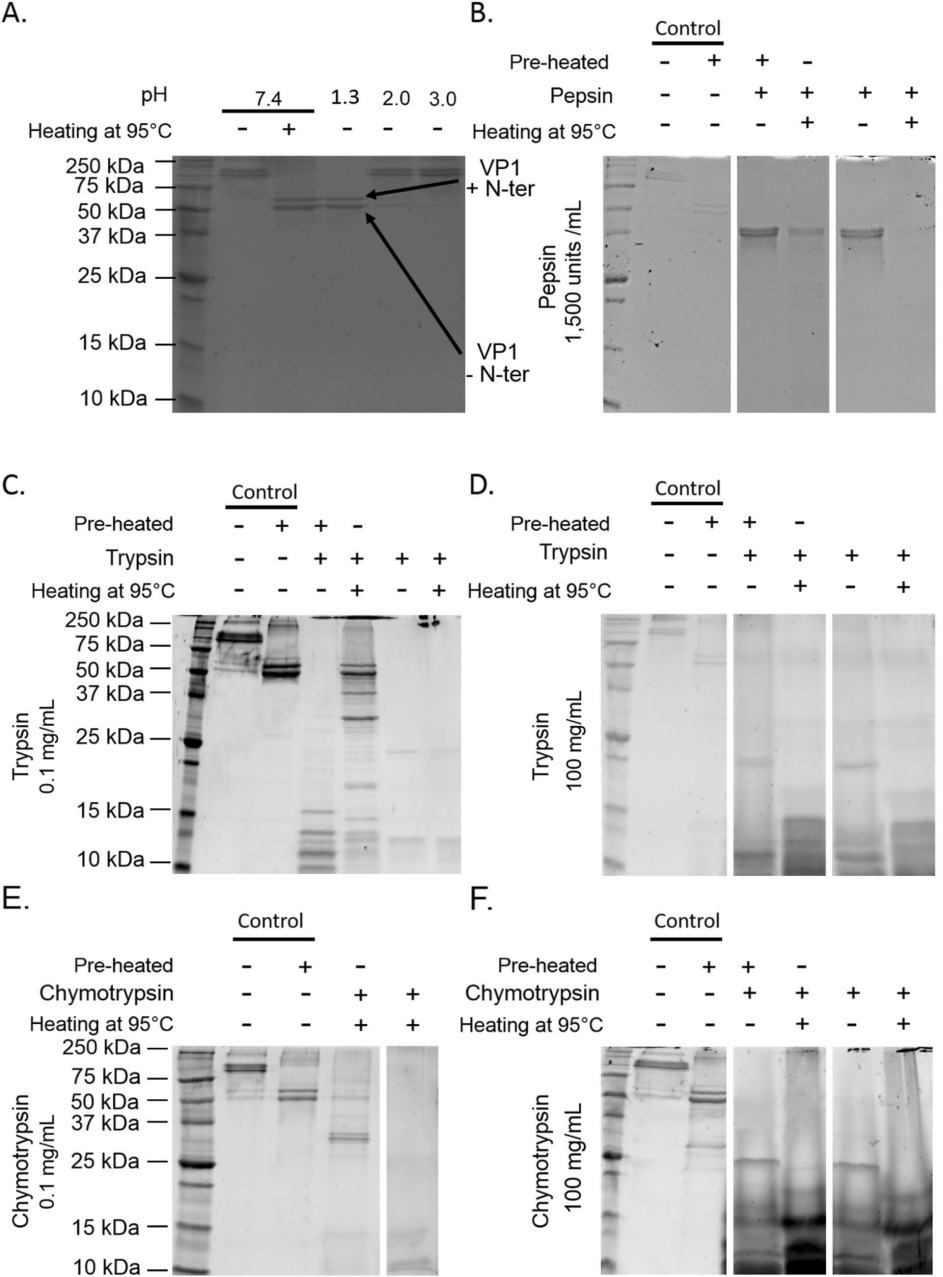
SDS-PAGE analyses of native and acid pH- or proteolytic enzyme-treated GII.4 VLPs. **A.** GII.4 VLPs after exposure to different acid pH conditions (7.4, 1.3, 2.0, and 3.0). **B.** GII.4 VLPs after exposure to 1,500 units/mL pepsin at pH 2.0. **C.** GII.4 VLPs after exposure to 0.1 mg/mL trypsin at pH 8.0. **D.** GII.4 VLPs after exposure to 100 mg/mL trypsin at pH 8.0. **E.** GII.4 VLPs after exposure to 0.1 mg/mL chymotrypsin at pH 8.0. **F.** GII.4 VLPs after exposure to 100 mg/mL chymotrypsin at pH 8.0. The term “Pre-heated” means that the VLPs were previously heated at 95 °C for 10 min to obtain VP1 prior to proteolytic enzyme treatments. The term “Heating at 95 °C” means that the VLPs or VP1 were heated at 95 °C for 10 min prior to SDS-PAGE analysis, to dissociate VLPs and remove secondary VP1 structures. The “ + ” and “-” signs indicate whether or not the treatment was applied. No sign means that there were no VLPs present. White spaces indicate the different parts of the same gels. Each condition was tested in triplicate.

The effects of each treatment on VP1 proteins were evaluated by examining modifications using SDS-PAGE under denaturing conditions. The results are shown in Fig. 2. At pH 7.4, the profile of native GII.4 VLPs displayed bands with a molecular weight (MW) of above 100 kDa (Fig. 2A). VP1s were expected to have a MW of around 60 kDa. A slightly reduced MW of around 57 kDa was also expected in VP1 lacking the N-terminal (Nt) region^37^. Applying a heat treatment of 95 °C to native VLPs for 10 min allowed the two expected VP1 bands to be observed using SDS-PAGE analysis. This suggests that, under denaturing conditions, SDS-PAGE disturbed the migration of VP1 to the expected MWs because of the incomplete dissociation of VP1 multimers and/or the conservation of some secondary structures. We will henceforth refer to such bands as “VP1 multimers.” Thus, the effects of different treatments, on GII.4 VLPs, both separately and in combination with heat treatment (referred to as “Heating at 95 °C” in Fig. 2) were investigated and then submitted to SDS-PAGE analysis. Different pH conditions were tested, with the following results: (i) similar profiles were obtained for native VLPs at pH 7.4 (i.e. the control) and VLPs treated at pH 3.0, and included the presence of “VP1 multimers”; (ii) a slight presence of VP1 and “VP1 multimers” was detected in VLPs treated at pH 2.0; and (iii) VP1 alone was observed in VLPs treated at pH 1.3. This indicates that heating or an acid pH of less than 2.0 dissociated or removed the “VP1 multimers”.

The effects of proteolytic enzymes (pepsin, trypsin, and chymotrypsin) on both the isolated form of VP1 protein and the form assembled into VLPs were investigated. As described above, the effect on isolated VP1 was studied by heating at 95 °C for 10 min prior to proteolytic enzyme treatments (referred to as “Pre-heated” in Fig. 2). The pepsin treatment resulted in the digestion of VP1, both isolated and assembled into VLPs (Fig. 2B). The bands observed at around 37 kDa correspond to pepsin with a MW of 34.6 kDa, which did not interfere with VP1. No digestion of VP1 was observed following the use of heat-inactivated pepsin (see Supplementary Information Fig. S1). The 0.1 mg/mL trypsin treatment completely digested the isolated VP1 and partially altered the VP1 assembled into VLPs (Fig. 2C). Increasing trypsin concentration to 100 mg/mL led to the complete digestion of VP1, whether isolated or assembled into VLPs (Fig. 2D). No digestion of VP1 was observed when using heat-inactivated trypsin (see Supplementary Information Fig. S1). Trypsin also appeared in the SDS-PAGE analysis, at a MW of 23.8 kDa. The 0.1 mg/mL chymotrypsin treatment also partially altered VP1 that were assembled into VLPs (Fig. 2E). Increasing chymotrypsin concentration to 100 mg/mL also led to a complete digestion of VP1, whether isolated or assembled into VLPs (Fig. 2F). Chymotrypsin was present at a MW of 27.8 kDa.

The combined results of all the treatments tested suggest the following conclusions: (i) treatments at pH 3.0 had no effect on the structure of VLP/VP1; (ii) treatments with 0.1 mg/mL trypsin or 0.1 mg/mL chymotrypsin or at a pH of less than 2.0 had partial effects; (iii) treatments with 1,500 units/mL pepsin, 100 mg/mL trypsin, or 100 mg/mL chymotrypsin induced VP1 digestion and VLP disruption.

### The effects of acid pH and proteolytic enzymes on GII.4 HuNoVs

None of the methods used on GII.4 VLPs were applicable to GII.4 HuNoVs because the quantity and purity of particles were much greater in VLPs than in HuNoVs. The DLS, SYPRO Orange probe, and SDS-PAGE methods were not sensitive enough to allow us to study the behaviour of HuNoVs when treated with an acid pH and proteolytic enzymes. We therefore drew on the results obtained from GII.4 VLPs, to determine whether proteolytic enzymes and acid pH could disrupt HuNoVs. The difference between disrupted and structured particles was determined based on the number of HuNoV gc from fully intact capsids. Thus, the HuNoV genome was systematically quantified using RNase treatment followed by RT-qPCR before and after proteolytic enzyme and acid pH treatments. However, it cannot be ruled out that degradation takes place at the level of the capsid without making the RNA accessible to RNases. Therefore, the thermal stability of HuNoVs was evaluated using the same approach, to determine whether the viral particles were disrupted by such treatments. Minor degradations may impact thermal stability.

**Figure 3:**
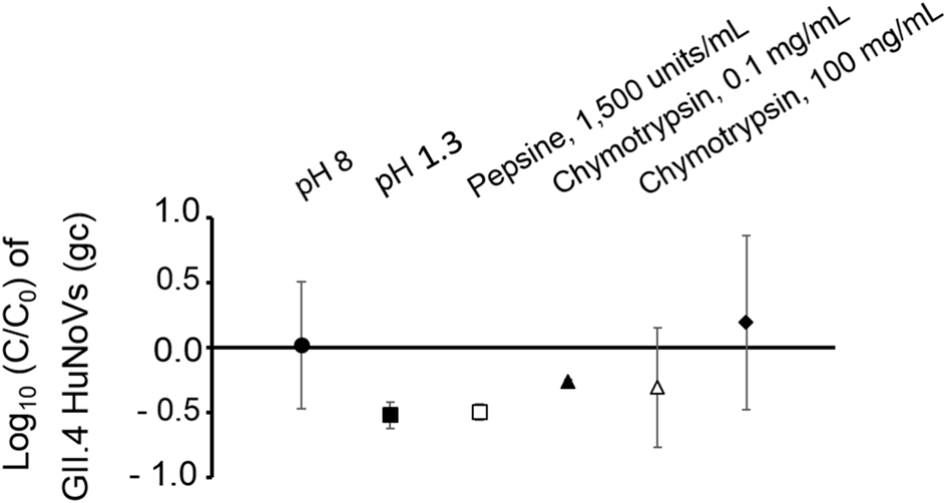
Decay of GII.4 HuNoVs after exposure to acid pH and proteolytic enzyme treatments. GII.4 HuNoV gc from fully intact capsids were quantified using RNase treatment followed by RNA amplification by RT-qPCR. The filled-in circle (●) represents native GII.4 HuNoV gc at pH 8.0. The filled-in square (■) and outlined square (□) represent GII.4 HuNoV gc treated at pH 1.3 alone and at pH 1.3 with 1,500 units/mL pepsin, respectively. The filled-in triangle (▲), outlined triangle (∆) and filled-in diamond (♦) represent GII.4 HuNoV gc after treatment at pH 8.0 with 0.1 mg/mL chymotrypsin, 100 mg/mL chymotrypsin, and 100 mg/mL trypsin, respectively. Each treatment was applied for 4 h at 37 °C. The log (C/C_0_) values were calculated by dividing the mean GII.4 HuNoV gc values following treatment by the mean native GII.4 HuNoV gc values at pH 8.0. Each data point is an average of at least two replicates. Error bars indicate standard deviations. The Mann–Whitney U test was used to compare groups^64^.

The effects of all these treatments on HuNoVs are shown in Fig. 3. In contrast with their effects on VLPs, there was no evidence that any of these treatments diminished the integrity of HuNoVs, when compared with native HuNoVs at pH 8.0 (Mann–Whitney U test, *p* > 0.01).

**Figure 4:**
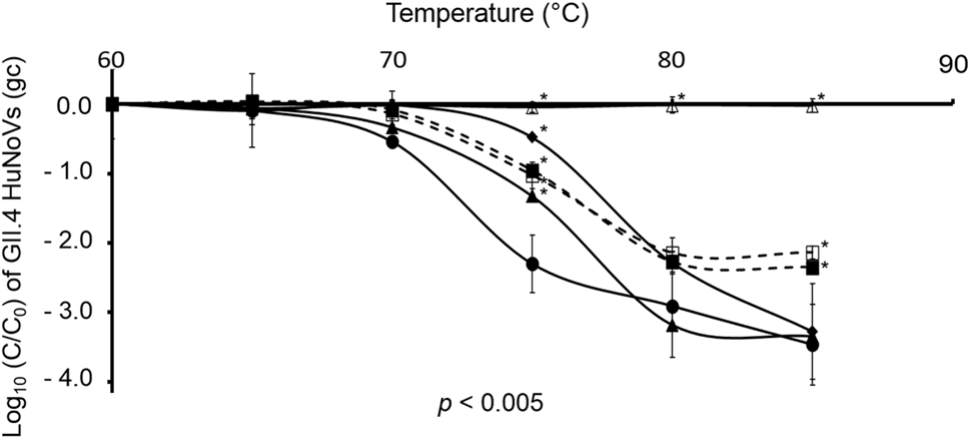
Decay of GII.4 HuNoV genomes after exposure to an acid pH and proteolytic enzyme treatments followed by heat treatment. GII.4 HuNoV gc from fully intact capsids were quantified using RNase treatment followed by RNA amplification by RT-qPCR. The filled-in circles (●, solid line) represent GII.4 HuNoVs at pH 8.0. The filled-in squares (■, dotted line) and outlined squares (□, dotted line) represent GII.4 HuNoV gc treated at pH 1.3 alone and at pH 1.3 with 1,500 units/mL pepsin, respectively. The filled-in triangles (▲, solid line), outlined triangles (∆, solid line) and filled-in diamonds (♦, solid line) represent GII.4 HuNoV gc at pH 8.0 after 0.1 mg/mL chymotrypsin, 100 mg/mL chymotrypsin, and 100 mg/mL trypsin, respectively. The acid pH and proteolytic enzyme treatments were applied for 4 h at 37 °C and followed by heat treatment at different temperatures. The log (C/C_0_) values were calculated by dividing the mean GII.4 HuNoV gc values after treatment by the mean GII.4 HuNoV gc values obtained at 60 °C. Each data point is an average of at least two replicates and error bars indicate standard deviations. An unpaired Student’s *t*-test and the Mann–Whitney U test were used to compare treated groups to the untreated one^64^.

As previously demonstrated with GII.4 VLPs, the thermal stability of HuNoVs could further explain the partial damage to capsids induced by proteolytic enzymes and their effects on the Tm values needed to disassemble the HuNoV capsid. HuNoV gc from fully intact capsids were therefore quantified at different temperature gradients, to evaluate the effects of acid pH and proteolytic enzyme treatments. The log_10_ (C/C_0_) values were determined by dividing the mean GII.4 HuNoV gc values after each proteolytic enzyme or acid pH treatment, following treatment at temperatures of 60–85 °C, by the mean GII.4 HuNoV gc values obtained after the same enzymatic treatment and treatment at 60 °C. The results are shown in Fig. 4. At pH 8.0, the native HuNoV capsids appeared to be highly disassembled at a Tm value of close to 75 °C. The Tm values of HuNoVs treated with proteolytic enzymes (chymotrypsin, trypsin, and pepsin) varied from 70 °C–80 °C. At 75 °C, the decay rates of GII.4 HuNoV gc treated with acid pH or proteolytic enzymes were significantly lower than those of native GII.4 HuNoVs at pH 8.0 (unpaired sample *t*-test, *p* < 0.005). Above this temperature, three profiles could be identified: (i) a decay rate similar to that of the native HuNoVs at pH 8.0 following treatment with 0.1 mg/mL chymotrypsin and 100 mg/mL trypsin (unpaired sample *t*-test or Mann–Whitney U test, *p* > 0.1); (ii) a decay rate lower than that of the native HuNoVs at pH 8.0 following both pH 1.3 treatment alone and pepsin treatment at pH 1.3 (unpaired sample *t*-test or Mann–Whitney U test, *p* < 0.002); and (iii) no decay rate for HuNoVs treated with 100 mg/mL chymotrypsin (unpaired sample *t*-test or Mann–Whitney U test, *p* < 0.002). It could, therefore, be concluded that the various treatments had no deleterious effects on the HuNoV capsids. Some of the treatments even seemed to play a protective role, which may be explained by their high enzyme content or by the aggregation of the capsid proteins, rendering the viral genome inaccessible to RNase.

In conclusion: none of the proteolytic enzymes or acid pH treatments led to a decrease in the Tm values needed to disassemble the GII.4 HuNoV capsids.

### Protective effect of HBGAs against proteolytic enzymes

**Figure 5:**
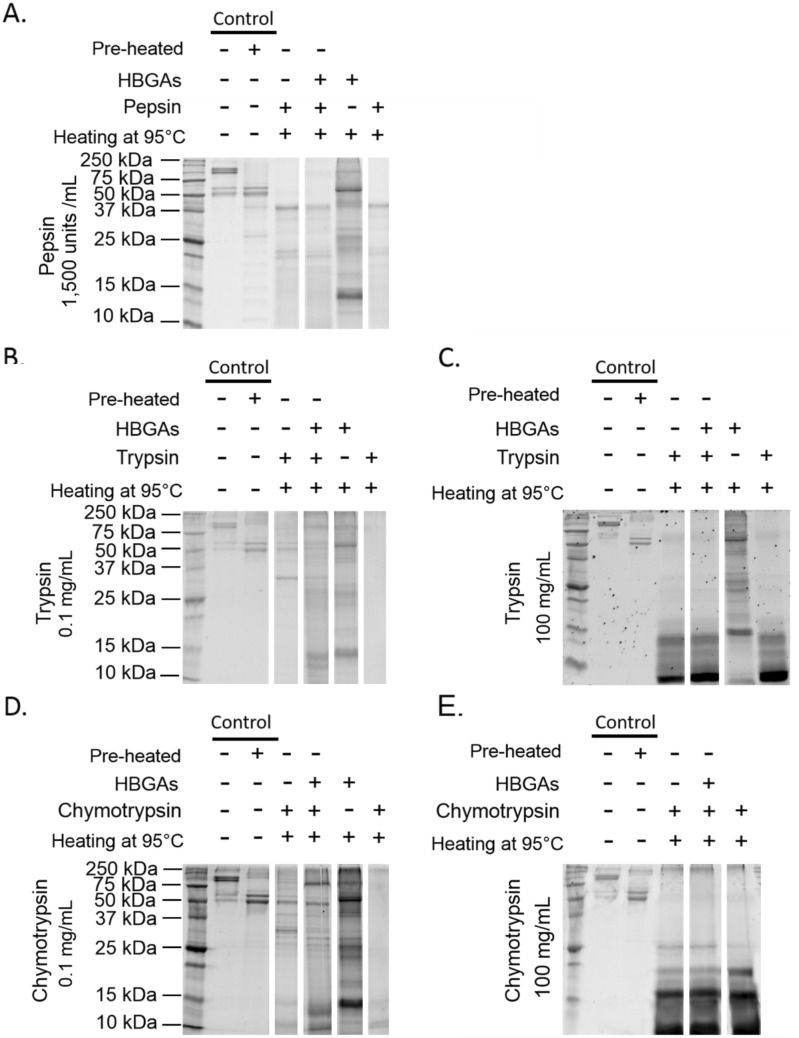
SDS-PAGE analyses of native and acid pH- or proteolytic enzyme-treated GII.4 VLPs with salivary HBGAs. **A** GII.4 VLPs with ALe^+^ after exposure to 1,500 units/mL pepsin at pH 2.0. **B** GII.4 VLPs with ALe^+^ after exposure to 0.1 mg/mL trypsin at pH 8.0. **C** GII.4 VLPs with ALe^+^ after exposure to 100 mg/mL trypsin at pH 8.0. **D** GII.4 VLPs with ALe^+^ after exposure to 0.1 mg/mL chymotrypsin at pH 8.0. **E** GII.4 VLPs with ALe^+^ after exposure to 100 mg/mL chymotrypsin at pH 8.0. The term “Pre-heated” means that the VLPs were previously heated at 95 °C for 10 min to obtain VP1 prior to the proteolytic enzyme treatments. The term “Heating at 95 °C” means that the VLPs were heated at 95 °C for 10 min just before SDS-PAGE analysis, to dissociate VLPs and remove secondary VP1 structures. The “ + ” and “−” signs indicate whether or not the treatment was applied. No sign means that no VLPs or HBGAs were present. White spaces indicate the different parts of the same gels. Each condition was tested in triplicate.

HBGA-binding’s potential protective effects on the viral capsid against proteolytic enzymes have only been evaluated for GII.4 VLPs, since these effects had no observable impact on HuNoVs. First, the HBGA-binding profile of GII.4 VLPs in 15 saliva samples was determined using the enzyme-linked immunosorbent assay (ELISA) approach (see Supplementary Information Fig. S2). The human saliva with the greatest relative affinity for GII.4 VLPs was selected and correlated with type ALe^+^ (“E” in Supplementary Information Fig. S2). Second, GII.4 VLPs were incubated with the ALe^+^ saliva prior to proteolytic enzyme treatments using pepsin, trypsin, or chymotrypsin. To determine whether HBGA-binding would have a protective effect on VLPs against these enzymes, SDS-PAGE analysis was used to investigate the stability of VP1. It can be assumed that HBGA-binding prevents the action of the enzymes if the relative intensities of VP1 bands of native VLPs and VLPs treated using the proteolytic enzymes are similar. The results of the SDS-PAGE analyses are shown in Fig. 5. Saliva containing HBGAs is rich in proteins, making some of the results difficult to interpret, particularly when VP1 was confused with unknown salivary proteins of the same MW (~ 58–59 kDa). At 0.1 mg/mL trypsin and chymotrypsin, the relative intensity of VP1 decreased in the same way as in the control group, without HBGA-binding. Moreover, at 1,500 units/mL pepsin, 100 mg/mL trypsin, or 100 mg/mL chymotrypsin, VP1 completely disappeared in the same way as it did in the control group, without HBGA-binding. These results mean that the specific binding interactions between GII.4 VLPs and HBGAs did not prevent enzymatic digestion of the viral capsids. We conclude that GII.4 VLPs were sensitive to all treatments and that HBGA-binding to VLPs does not have a protective effect on capsid stability under the conditions tested.

## Discussion

The role of HBGAs in HuNoV infection is still not perfectly understood, and progress is required to improve knowledge of their potential role. In research to date, HBGAs have been described to be involved in HuNoV infection^15,38,39^. While HBGAs were recently shown to be critical for infection of some HuNoV strains in the human intestinal enteroids system^40^, there is no evidence that HBGAs are the putative cellular receptor for HuNoVs.

Over the past decade, HBGAs have only been considered as co-receptors or co-factors promoting infection in host cells because of the hypotheses that HuNoV genotypes could (i) bind to cells (e.g. lamina propria, Brunner’s gland, and epithelial cells) independently of the presence of HBGAs^41,42^, (ii) cause acute gastroenteritis outbreaks regardless of the Lewis or ABO phenotypes of secretor individuals^43^ and (iii) infect non-secretor individuals^44,45^. To obtain a better understanding of the role of HBGAs in the entire host-cell infection process in humans, the protective role of these complex carbohydrates in the presence of various factors naturally found in the human digestive system was studied. The effects of acid pH and three proteolytic enzymes (pepsin, trypsin, and chymotrypsin) on capsid integrity in HuNoVs were investigated because interactions in the stomach and small intestine can occur before the virus reaches the primary target of infection and replication.

First, the effects of acid pH and of the three proteolytic enzymes described above on native GII.4 VLPs and GII.4 HuNoVs were tested. Complete disassembly of GII.4 VLPs—but not GII.4 HuNoVs—into VP1 was observed at an acid pH of less than 2.0. Capsid stability clearly differed between VLPs and HuNoVs belonging to the same genotype. The lesser stability of GII.4 VLPs compared to GII.4 HuNoVs was also demonstrated, following natural ageing at 20 °C^46^. This difference could have been due to the absence of VP2 and/or the lack of interactions between the viral genome and VP1^28,47^. These interactions have been reported to promote capsid stability in other viruses, such as F-specific RNA bacteriophages (MS2 and GA) as well as Triatoma virus (TrV), a picorna-like virus with a single-stranded positive-sense RNA genome and an icosahedral T = 3 structure similar to that of HuNoVs^48,49^.

The effects of pepsin, trypsin, and chymotrypsin were studied under optimal conditions. The optimal conditions for pepsin were a concentration of 1,500 units/mL in gastric fluid buffer, at a pH of 2.0 for VLPs and 1.3 for HuNoVs^50^. The optimal conditions for trypsin and chymotrypsin were 0.1 or 100 mg/mL at pH 8.0 in ammonium bicarbonate buffer^51^. Digestion of VP1 was observed after treatment with the three proteolytic enzymes, indicating the accessibility of cleavage sites. According to the ExPASy PeptideMass tool, 34 peptides, with MWs of 0.5–1.5 kDa should be obtained after pepsin treatment at pH 2.0. With trypsin and chymotrypsin, 28 peptides (of 0.5–5.1 kDa) and 48 peptides (of 0.5–2.1 kDa) respectively are expected following enzymatic digestion. Under the conditions tested, degradation of VP1 was confirmed by SDS-PAGE analyses, following proteolytic enzyme treatments. As expected, the VP1 obtained from native VLPs was totally digested after preheating to 95 °C. Nevertheless, VP1 was not completely cleaved when enzymatic digestion was performed using GII.4 VLPs, particularly at lower doses of proteolytic enzymes (i.e. 0.1 mg/mL). Some intact particles were detected by TEM observations, and some VP1 at a MW of 57 and 60 kDa was detected by SDS-PAGE analysis. Our results demonstrated that VP1 is more sensitive to enzymatic digestion in its isolated form than when assembled into GII.4 VLPs, as previously described in the case of GI.1 VLPs^52^. One peptide of 32 kDa was found in GI.1 VLPs and two peptides of 31 and 26 kDa in GII.3 and GII.6 VLPs respectively following trypsin treatments of 16–500 µg/mL^30–32,52^. Although complete digestion of VP1 at 62.5 °C and 500 µg/mL trypsin was observed using SDS-PAGE analysis, some authors could still observe VLPs using TEM^31,32^. The structure and stability of the HuNoV capsid therefore seems to be genotype dependent. It has recently been demonstrated that GII.4 VLPs have T = 4 icosahedral symmetry, whereas GI.1 and GI.7 VLPs have T = 3 icosahedral symmetry^53^. Thus, the intra- and inter-VP1 interactions of VP1 assembled into VLPs differ between HuNoV genotypes. This may explain why VP1 could be easily dissociated from VLPs in SDS-PAGE under denaturing conditions in some genotypes (i.e. GII.3 and GII.6) but not completely in GII.4 HuNoVs. The persistence of non-covalent multimers of VP1 at MWs above 100 kDa, which were only dissociated after heating at 95 °C or at an acid pH of less than 2.0 in our study, provides further evidence of the specificity of capsid structure at genotype level. Our results demonstrate that enzymatic cleavage of VP1 does not always reflect disruption of GII.4 VLPs because of the incomplete digestion of VP1 when assembled into VLPs. Complete disruption of GII.4 VLPs was obtained after treatments with trypsin and chymotrypsin concentrations higher than 100 mg/mL and pepsin concentrations higher than 1,500 units/mL.

While different degrees of enzymatic digestion were detected in GII.4 VP1 and VLPs, different behaviours were observed in native GII.4 HuNoVs following pepsin, trypsin, and chymotrypsin treatments. VLPs are described as structurally, antigenically, and morphologically similar to intact HuNoV particles^54^. Unlike GII.4 VLPs, in which evidence of partial or complete disruption was found following proteolytic enzyme treatments, no complete dissociation of GII.4 HuNoVs was obtained under the same conditions, regardless of the concentration of enzymes used. With the aim of evaluating whether these enzymes really could disrupt GII.4 HuNoV capsids, the thermal stability of the viral particles following gradual increases in temperature after proteolytic enzyme treatments was investigated. This provided several insights. Decay rates were similar in native GII.4 HuNoVs and GII.4 HuNoVs treated with 0.1 mg/mL chymotrypsin and 100 mg/mL trypsin after heating at all temperatures in the range except 75 °C; while in those treated with both a pH of 1.3 alone and with a pH of 1.3 and pepsin combined, less reduction in GII.4 HuNoV genomes was observed after heating to 75 °C and 85 °C. Conversely, no reduction in GII.4 HuNoV genomes was detected following 100 mg/mL chymotrypsin treatment over the whole heat treatment range, indicating the potential stability of the HuNoV capsids. These results suggest that chymotrypsin content was high enough to (i) provide a protective effect against heat degradation; and (ii) prevent HuNoV genomes from being accessible to RNases. The protective effect of a high chymotrypsin concentration could also be produced by treating HuNoVs with an acid pH and pepsin. This could be explained by the aggregation of viral particles which can maintain the integrity of particles^47^. Be that as it may, our results clearly indicate that GII.4 HuNoVs are more resistant to an acid pH and proteolytic enzyme digestion than GII.4 VLPs.

Second, the protective effect of HBGA-binding on GII.4 VLP capsids was investigated by using human saliva prior to proteolytic enzyme treatments. No similar investigation was conducted using GII.4 HuNoVs because there was no evidence of complete dissociation of the viral particles, induced by the same enzymes. At low doses of trypsin and chymotrypsin (0.1 mg/mL), SDS-PAGE analysis detected no difference between samples with and without HBGA-binding to VLPs. At higher doses of trypsin or chymotrypsin (100 mg/mL), and at 1,500 units/mL pepsin, VLPs were totally disrupted even in the presence of HBGAs. Thus, we found no evidence that ALe^+^ human saliva provided a protective effect on capsid integrity in GII.4 VLPs against these proteolytic enzymes. Several assumptions can be drawn on the basis of these observations. The first is that the cleavage sites of pepsin, trypsin, and chymotrypsin are not located in the regions surrounding the HBGA-binding site in subdomain P2 of VP1. The second is that the steric hindrance caused by HBGAs was not sufficient to prevent capsid digestion by these proteolytic enzymes. However, it is important to bear in mind some methodological constraints on investigating the potential influence of HBGA-binding on the maintenance of capsid integrity. For example, it was difficult to use SYPRO Orange probe to study the Tm values of VLPs. The abundance of proteins in human saliva and the addition of the proteolytic enzymes increased the background noise and hindered the reliable determination of Tm values. Given that proteolytic enzymes and an acid pH of less than 2.0 can disrupt VLPs, the present study demonstrated, for the first time as far as we know, that human saliva containing HBGAs does not influence the stability of the capsid when exposed to factors naturally found in the human digestive system. However, that bile salts or divalent cations could promote capsid stability has not been ruled out. There is ample evidence that they enhance the replication of HuNoVs using human intestinal enteroids but also potentially improve HBGA-binding to HuNoVs^8,55,56^. The protective effect of human gut microbiota could also be involved because it has recently been shown that some enteric bacteria (e.g. *Enterobacter cloacae*) bind HuNoVs through the H-like carbohydrates present on the bacterial surface^57,58^.

Finally, we demonstrated the differences between the behaviours of GII.4 VLPs and GII.4 HuNoVs when exposed to different treatments (acid pH and proteolytic enzyme treatments). There was no evidence of a protective effect of HBGAs on GII.4 VLPs. Further research is required to elucidate how HBGA-binding contributes to capsid stability and to human infections.

## Materials and methods

### Purification of GII.4 VLPs and GII.4 HuNoVs

GII.4 VLPs (Cairo 4 strain, a 2007 Osaka variant) were obtained using the baculovirus-expressed VP1 system and purified using a discontinuous sucrose gradient, as previously described by De Rougemont^59^. The quantity of proteins was determined using a NanoDrop 2000 spectrophotometer (Thermo Fisher Scientific, Waltham, MA, USA). GII.4 VLPs were prepared at a final concentration of 1 mg/mL with a TNC buffer (10 mM Tris-HCl, 140 mM NaCl, 10 mM CaCl_2_, pH 7.4). Aliquots were stored at − 80 °C, and extemporaneously thawed as needed.

The GII.4 HuNoVs were genotyped from human stools^60^, kindly provided by the University Hospital of Nancy, France. The purification, extraction, and genome quantification of GII.4 HuNoVs were performed using the methods described previously^46^ (see Supplementary Information Method). The purified HuNoV suspensions were stored in darkness at +4 °C at a final concentration of 10^7^ gc/mL until needed. The total protein content of the HuNoV suspensions was measured using a NanoDrop 2000 spectrophotometer at a final concentration of 350 µg/mL.

### Acid pH and proteolytic enzyme treatments of GII.4 VLPs and GII.4 HuNoVs

Suspensions of GII.4 VLPs were diluted in simulated gastric fluid buffer (25 mM NaHCO_3_, 12 mM KCl, 40 mM NaCl, 6 mM CaCl_2_)^50^ at pH values ranging from 1.3 to 3.0 or in ammonium bicarbonate buffer (50 mM NH_4_HCO_3_, pH 8.0). GII.4 VLPs were prepared at a final concentration of 300 µg/mL. For the acid pH treatments, 4 µL of the simulated gastric fluid buffer were added to 10 µL of GII.4 VLPs at 300 µg/mL. For the proteolytic enzyme treatments, 10 µL of GII.4 VLPs at 300 µg/mL was treated with 4 µL of proteolytic enzymes at 37 °C for 4 h. Pepsin (Promega, 02195367) was used at a concentration of 1,500 units/mL (i.e. 600 µg/mL) in simulated gastric fluid buffer at pH 2.0 for VLPs and pH 1.3 for HuNoVs. Trypsin (Sigma, T4799) and chymotrypsin (Fisher Scientific, 10568362) were used at concentrations of 0.1 and 100 mg/mL in ammonium bicarbonate buffer.

The protocol developed for proteolytic enzyme treatments on GII.4 VLPs was used on the purified GII.4 HuNoVs. To estimate the quantity of potentially fully intact capsids, the purified GII.4 HuNoV suspensions were treated with RNase A (Thermofisher, EN0531) as described by Brié et al., with some modifications^61^. An internal control was added to the GII.4 HuNoV suspensions to confirm the action of RNase. Briefly, 10 µL of MS2 bacteriophage RNA genome at a concentration of 10^6^ gc/mL was added to 10 µL of GII.4 HuNoV suspension. Then, 8 μL of the proteolytic enzyme (pepsin, trypsin, or chymotrypsin) was added and the mixture was incubated at 37 °C for 4 h. RNase was added to achieve a final concentration of 100 μg/mL. The results were considered valid if the RNA genomes of MS2 bacteriophages was not detected by RT-qPCR (see Supplementary Information Method). The number of HuNoV gc in the native suspension was quantified using RT-qPCR at pH 8.0 and 37 °C (C_0_) and following acid pH or proteolytic enzyme treatments at 37 °C (C). The experiments involving GII.4 VLPs and GII.4 HuNoVs were at minimum duplicated, under the same conditions.

### TEM for GII.4 VLPs

TEM was used to observe GII.4 VLPs both with and without acid pH or proteolytic enzyme treatments, after negative staining with phosphotungstic acid, as previously described^46^. TEM is considered to provide a semi-quantitative estimation of the number of particles. Pictures at a magnification of ×15,000 were obtained, to provide a representative field permitting an estimation of the number of particles, and at a magnification of ×27,500 to provide a more precise overview of the morphology and size of particles. When assessing each acid pH, proteolytic enzyme treatment, and magnification factor, at least 4 representative pictures were taken into account. The mean number of particles was determined by visual inspection and the size was measured using ImageJ software (Java 1.8.0).

### DLS for GII.4 VLPs

Only the GII.4 VLPs treated with 0.1 mg/mL and 100 mg/mL trypsin and chymotrypsin were studied using this method. At the end of the proteolytic enzyme treatments, the reaction was diluted by the addition of 1 mL of 0.1× PBS (13.7 mM NaCl, 0.27 mM KCl, 1.0 mM Na_2_HPO_4_, 0.18 mM KH_2_PO_4_, pH 7.4). Size measurements were made using a Zetasizer Nano ZS instrument (Malvern Instruments), as described by Brié et al.^61^. Diameter measurements of GII.4 VLPs were obtained using at least two independent experiments and each hydrodynamic diameter measurement was performed in triplicate.

### Differential scanning fluorimetry for GII.4 VLPs and GII.4 HuNoVs

The thermal stability of GII.4 VLPs was studied as previously described^62^, with slight modifications. Tubes were filled with 5 µL of 150 mM Tris-HCl pH 8.0, 5 μL of VLPs, with or without acid pH or proteolytic enzyme treatments, and 13 µL of distilled water. Finally, 2 µL of 62X SYPRO Orange solution were added. The tubes were prepared on ice and a control prepared under the same conditions was added to each experiment. Equilibration was performed and fluorescence intensity recorded as previously described^62^. This allowed the Tm values that corresponded to the temperatures at which viral hydrophobic domains became accessible to the hydrophobic probe to be determined. For each condition tested, the Tm values of GII.4 VLPs were obtained in triplicate.

The method used on GII.4 VLPs could not be applied to GII.4 HuNoVs because the viral content of the purified stools was insufficient. A new procedure was therefore implemented to determine the temperature needed to disassemble the GII.4 HuNoV capsids. Following proteolytic enzyme treatments, the samples were placed in a Veriti Thermal Cycler (Applied Biosystems, Foster City, CA, USA) apparatus and heated at different temperatures (i.e. 37 °C, 60 °C, 65 °C, 70 °C, 75 °C, 80 °C, and 85 °C) for 10 min. RNase A treatment was then applied to a final concentration of 100 μg/mL. Finally, the viral genomes of MS2 bacteriophages and GII.4 HuNoVs were extracted and quantified using RT-qPCR as described above and in the Supplementary Information. The quantity of HuNoV gc was determined in the initial viral suspensions, following treatment at 60 °C (C_0_), and at the different temperatures tested using the same treatment (C). For each sample, at least two analyses of HuNoVs were performed.

### SDS-PAGE for GII.4 VLPs

GII.4 VLPs treated with an acid pH or proteolytic enzymes were analysed using SDS-PAGE. The effects of enzymatic treatments on VP1 and VLPs were studied. VP1 was obtained from VLPs by heating them in a thermomixer (Thermomixer comfort; Eppendorf, Hamburg, Germany) at 95 °C for 10 min, then placing them on ice.

Following the proteolytic enzyme treatments, GII.4 VLPs were heated for 10 min at 95 °C in order to compare the effects of the treatments on VP1 and VLPs.

GII.4 VLPs were then denatured using SDS and dithiothreitol (DTT) conditions, as previously described, with slight modifications^62^. Aliquots of 20 µL of each denatured sample were loaded onto a discontinuous polyacrylamide gel (4% staking gel, 13% resolving gel). Standard size markers, protein migration, and gel staining were used under the same conditions as previously described^62^. Analyses of each sample were performed in triplicate.

### Salivary HBGA types

Saliva samples from healthy adult volunteers were collected. The saliva samples used in this study were previously described, with the approval of the Nantes University Hospital Review Board (study number BRD02/2-P)^59^, and informed consent was obtained from all donors. All the experiments using saliva were performed in accordance with the relevant guidelines and regulations. The human saliva was boiled and centrifuged, as previously described^46^. Supernatants were stored at − 20 °C until use. The presence of A, B, and O blood group antigens and Lewis antigens in saliva samples at a dilution of 1:1000 in 1× PBS was determined by ELISA, as previously described^63^.

### Binding of GII.4 VLPs to saliva samples: HBGA-binding ELISA

HBGA-binding ELISA was performed using the method previously described^46^, with slight modifications. The modifications involved a negative control. Briefly, saliva samples were treated with 100 mM sodium periodate (prepared in a 50 mM sodium acetate buffer at pH 5) for 40 min at room temperature, and then incubated for 10 min with 1% glycine in 1× PBS.

Optical density values were obtained at 450 nm (OD_450_) and the results were analysed as previously described^46^. All the samples were analysed in duplicate. Results, expressed in percentages, were calculated by taking the strongest adhesion of GII.4 VLPs as a reference.

### Interaction with human saliva prior to proteolytic enzyme treatments

Ten µL of native GII.4 VLPs diluted to 300 µg/mL in a buffer of proteolytic enzyme were incubated with 10 µL of ALe^+^ saliva sample at 37 °C for 1 h. Then, 8 µL of proteolytic enzymes (i.e. 1,500 units/mL pepsin, 0.1 or 100 mg/mL trypsin, 0.1 or 100 mg/mL chymotrypsin) were added and the samples were stored at 37 °C for 4 h. All results were analysed in triplicate, using SDS-PAGE.

### Statistical analysis

All statistical analyses were performed using R statistical software (Rx64 v.3.5.3)^64^. A Shapiro–Wilk test with an alpha level of 0.01 was run, to test the normality of the data. Student’s paired and unpaired t-tests were used for dependent and independent data respectively, following a normal distribution. Wilcoxon signed-rank and Mann–Whitney U tests were used for dependent and independent data respectively with non-normal distribution. In all tests, the significance level was set to 0.01.

## Supplementary information

Supplementary Information

## Data Availability

All data analysed in this study are included in this manuscript and the accompanying supplementary information file.
