# Supplementary material for: The effect of proteolytic enzymes and pH on GII.4 norovirus, during both interactions and non-interaction with Histo-Blood Group Antigens
Source: Sci Rep. 2020 Oct 21;10:17926. doi: 10.1038/s41598-020-74728-z (PMC7578656; doi:10.1038/s41598-020-74728-z)
Supplement: Supplementary file 1 — Supplementary Information [file 41598_2020_74728_MOESM1_ESM.docx]

**The effect of proteolytic enzymes and pH on GII.4 norovirus, during both interactions and non-interaction with Histo-Blood Group Antigens**

**Manon Chassaing^1,2^, Maëlle Robin^1^, Julie Loutreul^1^, Didier Majou^3^, Gaël Belliot ^4,5^, Alexis de Rougemont ^4,5^Nicolas Boudaud^1^ and Christophe Gantzer^2*^**

^1^Actalia, Food Safety Department, F-50000 Saint-Lô, France.

^2^University of Lorraine, CNRS, LCPME, F-54000 Nancy, France.

^3^ACTIA, F-75231 Paris Cedex 05, France.

^4^ National Reference Centre for Gastroenteritis Viruses, Laboratory of Virology, University Hospital of Dijon, F-21000, France.

^5^ UMR PAM A 02.102 Procédés Alimentaires et Microbiologiques, Université de Bourgogne Franche-Comté/AgroSup Dijon, F-21000, France.

*Corresponding author: christophe.gantzer@univ-lorraine.fr


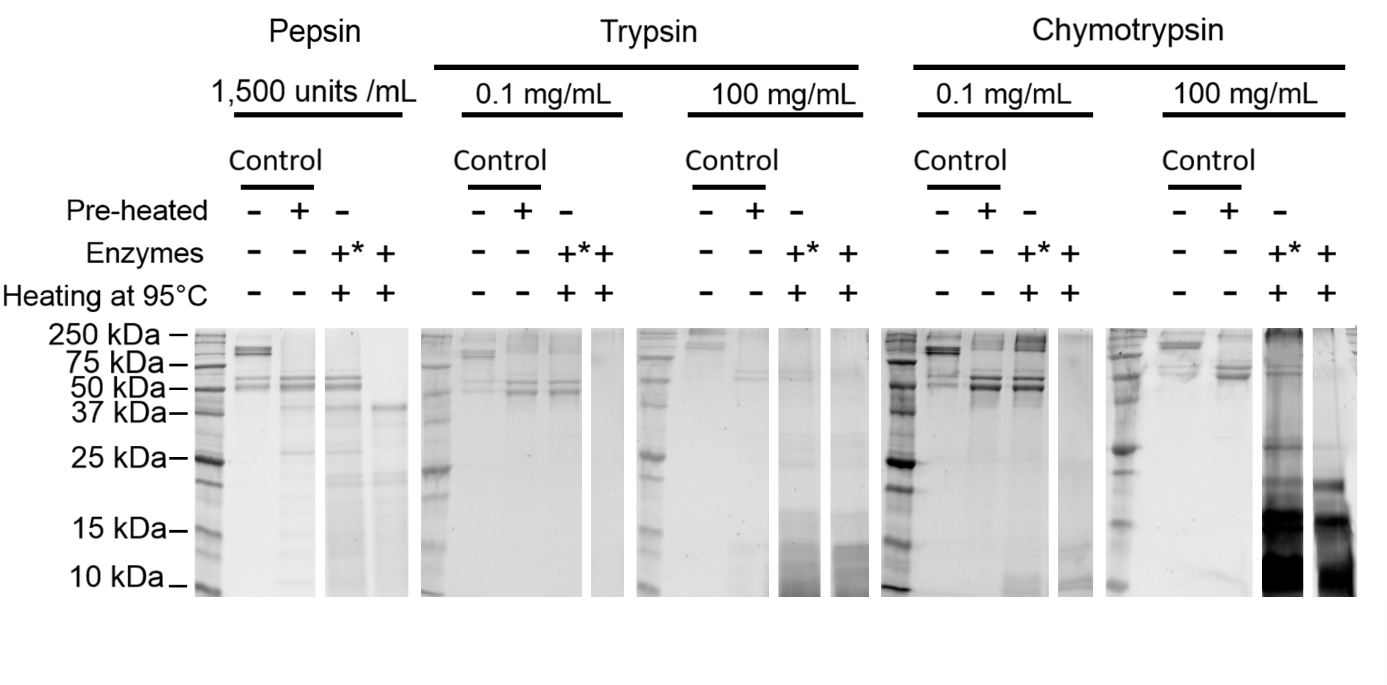


**Supplementary Figure S1**. **SDS-PAGE analysis of native and inactivated proteolytic enzyme-treated GII.4 VLPs.** The term “Pre-heated” means that the VLPs were previously heated for 10 min at 95°C, to obtain VP1 prior to the proteolytic enzyme treatments. The term “Enzymes” refers to the proteolytic enzymes used (1,500 units/mL pepsin at pH 2.0; 0.1 and 100 mg/mL trypsin; and 0.1 and 100 mg/mL chymotrypsin at pH 8.0). The term “Heating at 95°C” means that the VLPs or VP1 were heated for 10 min at 95°C prior to SDS-PAGE analysis to dissociate the VLPs and remove secondary VP1 structures. The “+” and “-” signs indicate whether or not the treatment was applied. *Enzymes were heated at 95°C for 10 min to inactivate them prior to use in the treatment of VLPs. No sign means that there were no VLPs present. White spaces indicate the different parts of the same gels. Each condition was tested in triplicate.


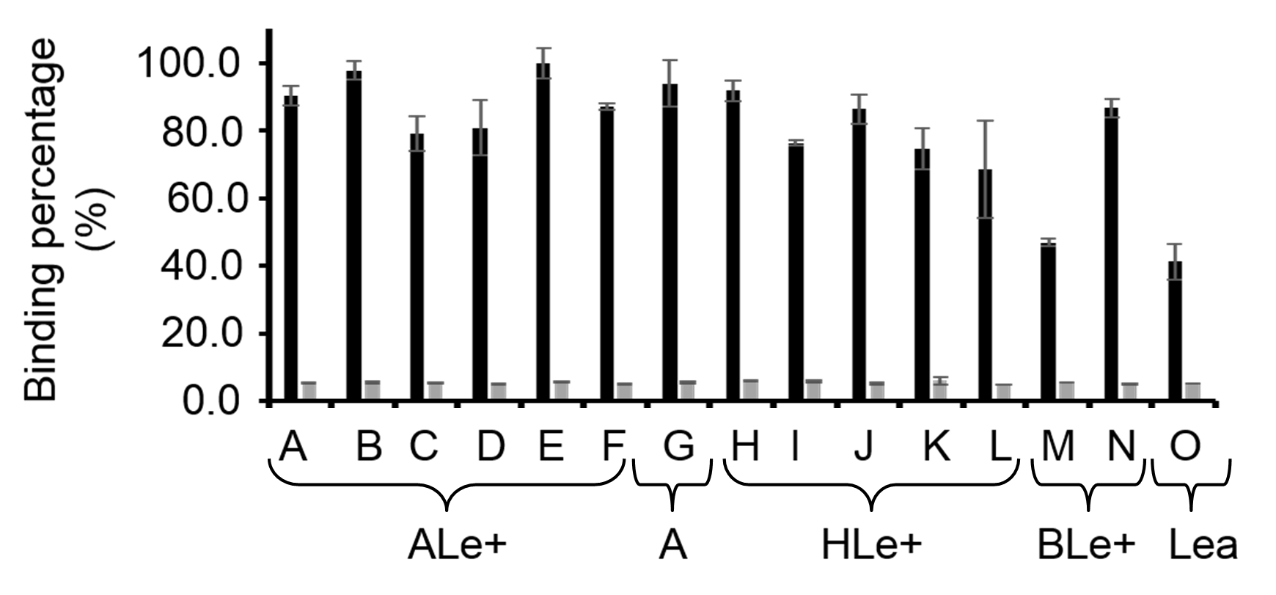


**Supplementary Figure S2.** **Binding profiles of GII.4 VLPs to all human saliva samples**. Results were obtained at 1 µg/mL of GII.4 VLPs in 1X PBS solution using HBGA-binding ELISA and are expressed as a percentage of HBGA-binding to VLPs. The 100% corresponds to the higher HBGA-binding of GII.4 VLPs. Black and grey bars indicate HBGA-binding to GII.4 VLP capsids, using untreated and sodium periodate-treated saliva samples, respectively. The uppercase letters indicate the saliva type used and the lowercase letters indicate HBGA type as previously characterized. Each data point represents the OD_450_ mean of two replicates and error bars indicate standard deviations.

**Supplementary Method.**

**Preparation of GII.4 HuNoV samples**^1^**.**

One g of fecal suspensions of GII.4 HuNoVs was added to 10 mL of 150 mM PBS solution at pH 7.4. Samples were clarified by addition of 1/3 (v/v) of chloroform, vortexed for 60 sec and centrifuged at 2,500 x *g* for 5 min. Supernatant was submitted to a second clarification step as described previously. The clarified samples were purified by dialysis using Float-A-Lyzer G2 with Biotech Cellulose Ester membranes (MWCO: 100 kD, volume: 1 mL, Spectra/Por, Spectrum Laboratories, CA, USA). The membranes were dialyzed in a tank containing 10 L of 10 mM PBS at 4°C overnight with gentle stirring. Dialysate samples were recovered and filtered using a 0.22 µm cellulose acetate membrane. After each step, the loss of HuNoV genomes was controlled. The viral genomes from 100 µL of purified HuNoV suspensions were extracted directly using the NucliSENS easyMAG kit (bioMérieux, Marcy-l’Etoile, France). The HuNoV genomes were detected using an RNA UltraSense One-Step quantitative RT-PCR system (Life Technologies, Carlsbad, CA, USA), according to the ISO 15216-1 standard recommendations^2^. Cq values were determined using CFX Maestro software (BioRad, Hercules, CA, USA). Quantification of HuNoV gc was performed using a standard curve of plasmids with a concentration range of 5 to 10^5^ gc/reaction mixture. Until use, the purified HuNoV suspensions were stored at 4°C in the dark at a final concentration of 10^7^ gc/mL.

**Production of MS2 phages and extraction of RNA genomes.**

Production and extraction of MS2 phage was performed according to the procedure described by Brié et al. (2018)^3^. Briefly, Replication of MS2 phage (ATCC, 15597-B1) was done according to the standard procedure (ISO 10705-1, 2001)^4^ (without the chloroform step) using *Escherichia coli* Hfr K12 (ATCC, 23631) as host cells. Prior to the RT-qPCR assays, extractions of viral RNA (50 μL) were performed using the QIAamp viral RNA kit (Qiagen, Hilden, Germany) according to the manufacturer’s recommendations.

**MS2 RT-qPCR detection.**

Viral MS2 RNA was detected using RNA UltraSense One-Step quantitative RT-PCR System (Life Technologies, Carlsbad, CA, USA). For reverse transcription (RT) and qPCR, 2 µL of extracted viral agent was mixed with 4 µL of 5X buffer; 0.5 µL of 0.5 µM forward 5’-TCGATGGTCCATACCTTAGATGC-3’; 0.5 µL of 0.5 µM reverse 5’- ACCCCGTTAGCGAAGTTGCT-3’; 0.4 µL of 0.4 µM probe (5’-FAM–CTCGTCGACAATGG–MGBNFQ-3’); 0.5 µL of ROX; and 0.75 µL of enzyme mix (including SuperScript III RT, Platinum Taq DNA Polymerase, and RNaseOUT Ribonuclease Inhibitor) in a 20 µL reaction volume. The RT step was performed for a duration of 30 min at 50°C. qPCR was then performed at 95°C for 5 min, followed by 45 cycles of 15 s at 95°C and 40 s at 58°C. Results were obtained using the StepOnePlus v2.3 software (Applied BioSystem, Foster City, CA, USA). The primers and probe used in the RT-qPCR were as described by Ogorzaly and Gantzer (2007)^5^. Two negative controls were included in each experiment.

**REFERENCES**

1. Robin, M. *et al.* Effect of natural ageing and heat treatments on GII.4 norovirus binding to histo-blood group antigens. *Sci. Rep.* **9**, 1–11 (2019).
2. ISO 15216-1. Microbiology of the food chain - Horizontal method for determination of hepatitis A virus and norovirus using real-time RTPCR- Part 1: Method for quantification (2017).
3. Brié, A. *et al.* The impact of chlorine and heat on the infectivity and physicochemical properties of bacteriophage MS2. *FEMS Microbiol. Ecol.* **94,** 106 (2018).
4. ISO 10705-1. Water Quality: Detection and enumeration of bacteriophages. Part 1: Enumeration of F-specific RNA bacteriophages (2001).
5. Ogorzaly, L. & Gantzer, C. Erratum to “Development of real-time RT-PCR methods for specific detection of F-specific RNA bacteriophage genogroups: application to urban raw wastewater." *J. Virol. Methods* **143**, 122 (2007).
